# Seasonal Diversity and Occurrence of Filamentous Fungi in Smallholder Dairy Cattle Feeds and Feedstuffs in South Africa

**DOI:** 10.3390/jof8111192

**Published:** 2022-11-11

**Authors:** Oluwasola Abayomi Adelusi, Sefater Gbashi, Janet Adeyinka Adebiyi, Rhulani Makhuvele, Adeola Oluwakemi Aasa, Oluwaseun Mary Oladeji, Minenhle Khoza, Sheila Okoth, Patrick Berka Njobeh

**Affiliations:** 1 Department of Biotechnology and Food Technology, Faculty of Science, University of Johannesburg, Doornfontein Campus, Johannesburg P.O. Box 17011, South Africa; 2 Department of Biological sciences, University of Nairobi, Nairobi P.O. Box 30197-00100, Kenya

**Keywords:** dairy feed, fungi, mycotoxins, seasonal variation

## Abstract

This study investigated 65 (35 in summer and 30 in winter) smallholder dairy cattle feeds from Free State and Limpopo provinces in South Africa from 2018 to 2019 for fungal contamination and assessed the impacts of seasonal variation on fungal contamination levels, isolation frequency, and diversity. Samples were examined for fungal contamination using macro- and microscopic approaches, and their identities were confirmed by molecular means. A total of 217 fungal isolates from 14 genera, including *Aspergillus*, *Fusarium*, and *Penicillium*, were recovered from feeds from both seasons. The most prevalent fungal species recovered were *A. fumigatus* and *P. crustosum*. Mycological analyses showed that 97% of samples were contaminated with one or more fungal isolates, with the summer fungal mean level (6.1 × 10^3^ to 3.0 × 10^6^ CFU/g) higher than that of feeds sampled during winter (mean level: 1.1 × 10^3^ to 4.1 × 10^5^ CFU/g). Independent sample *t*-test revealed that the isolation frequencies of the genera *Aspergillus* and *Fusarium* were significantly (*p* ≤ 0.05) higher in summer than winter, while *Penicillium* prevalence in both seasons was not statistically (*p* > 0.05) different. Furthermore, the Shannon–Weiner diversity index (H′) revealed a higher fungal diversity in summer (H′ = 2.8) than in winter (H′ = 2.1). This study on fungal contamination could be used for future fungal control and mycotoxin risk management in South Africa.

## 1. Introduction

Milk and milk products play an essential role in human nutrition due to a wide range of essential nutrients present in them, which are relevant to human and animal health [1,2]. In South Africa (SA), cattle milk production occurs on both smallholder and commercial farms. Ojango et al. [3] defined smallholder dairy farms as less established and poorly resourceful farms with fewer than 10 dairy cattle reared on small land sizes (between 0.2 and 4 hectares) compared to commercial dairy farms, which are capital intensive and well-developed with large herd sizes. This dairy sub-sector owns over 40% of the total 1.4 million dairy cattle in the country [4], with most smallholder dairy cattle farms situated in Free State and Limpopo provinces of SA. However, one of the most significant impediments to smallholder dairy cattle productivity in the country is shortage of high-quality feeds [5]. Dairy complete feeds and feed ingredients in SA are prone to contamination from various sources such as environmental pollution and activities by microorganisms, pests, and insects [6,7]. Among feed contaminants, contamination with microbes, particularly filamentous fungi, are a severe concern for dairy cattle production due to attendant mycotoxins they produce, which cause diseases and death in animals and humans.

A recent study by Schaarschmidt and Carsten [8] showed that these toxins are difficult to eliminate completely even during feed ingredient production and feed processing, leading to their “carry-over” from dairy feeds to milk and milk products [9]. Fungi contaminating dairy cattle feeds are divided into two groups viz: field fungi that invade feed ingredients and produce mycotoxins in the field, for example, *Fusarium* and *Alternaria* spp., and storage fungi that colonise feeds and produce toxins during storage, such as *Penicillium* and *Aspergillus* spp. Among these fungal genera, *Penicillium*, *Aspergillus*, and *Fusarium* are generally known as the most challenging contaminants of dairy cattle feeds and feed ingredients in SA [10,11]. Fungal colonisation of food and feed may occur under favourable conditions such as temperature, water activity, carbon dioxide, oxygen availability, and mechanical damage to the host [12,13]. These conditions, coupled with poor agricultural practices, are responsible for the prevalence of toxigenic fungi and mycotoxins in agricultural products, including dairy feed in the country [14]. Furthermore, mycotoxins, including aflatoxins (AFB_1_, AFB_2_, AFG_1_, and AFG_2_) formed by *Aspergillus* spp., especially *Aspergillus flavus* and *A. parasiticus*, zearalenone (ZEN) produced by *Fusarium oxysporum*, *F. equiseti*, *F. graminearum*, *F. culmorum* and *F. incarnatum*, fumonisins (FB_1_ and FB_2_) produced by *F. verticillioides* and *F. proliferatum*, deoxynivalenol (DON) formed by F. culmorum and F. graminearum, as well as ochratoxins (OTA) formed by the genera *Aspergillus* (*A. niger*, *A. ochraceus* and *A. carbonarius*) and *Penicillium* (*Penicillium verrucosum*), are common mycotoxins regularly reported in South African dairy cattle feeds and feed ingredients [15]. The toxicity induced by these fungal toxins in humans and animals includes genotoxicity, neurotoxicity, hepatotoxicity, nephrotoxicity, and immunosuppression [16]. Inhalation or ingestion of spores from mouldy feeds and feedstuffs by dairy cattle can cause severe illnesses generally termed “mycosis”. Examples in dairy cattle include mycotic abortions, mastitis, haemorrhagic bowel syndrome (HBS), and pneumonia [17,18]. Fungal infection in dairy cattle is of great economic importance due to reduced milk production, delay conception, infertility, abortion, direct loss of calves, as well as costs incurred on drugs and veterinary aids [19]. Humans are not exempted from being infected with associated fungal diseases. In SA, around 3885 cases of invasive aspergillosis, a disease caused by human exposure to spores of the *Aspergillus* genus, are recorded each year, most of which are potentiated primarily by syndemics of tuberculosis, HIV, and poverty across the nation [20]. Due to the growing global awareness of food safety and various factors such as climate change, which trigger fungal contamination of agricultural commodities, regular evaluation of fungal contamination in feed and food is required to ensure a healthy food supply along the food chain.

Identification of fungi as previously performed via conventional method has often been based on similarities in morphological characteristics between species as well as variability and mutation occurrence in cultures. Nonetheless, morphological identification can be quite difficult and inadequate for fungal characterisation due to the shortage of mycologist experts, cryptic speciation, hybridisation and convergent evolution [21]. Advances in molecular techniques have therefore made it possible to further elucidate differences between fungal species within a shorter time based on genetic diversity [22]. Unfortunately, little is known about the profile of fungal species contaminating dairy feeds in SA, as well as the impact of seasonal variation on their diversity. Therefore, this study aims at identifying the mycoflora contaminating smallholder dairy cattle feeds and feed ingredients in Limpopo and Free State provinces of SA and to evaluate the species diversity during different seasons (winter and summer) between 2018 and 2019.

## 2. Materials and Methods

### 2.1. Study Sites

The two agroecological distinct provinces of SA selected for this study were Limpopo and Free State. Limpopo province is located in the country’s far north, with warmer arid to semiarid or sub-humid tropical climates, while Free State is located in the central part of the country and has a subtropical, cooler arid to semiarid environment. Registered active smallholder dairy cattle owners who were beneficiaries of the Agricultural Research Council (ARC) developmental programmes in the Sekhukhune and Vhembe districts (Limpopo) and the Phuthaditjhaba district (Free State) were chosen for this study. The selection of the two provinces was based on differences in agroecological zones, the vast number of smallholder dairy farms found there, and feed availability.

### 2.2. Sample Collection

Ten smallholder dairy cattle farms in the Vhembe and Sekhukhune districts of Limpopo province and eleven farms in the Phutaditjaba region of Free State in SA were randomly sampled. The number of feeds collected from each farm ranged from one to four, depending on the type of feed available. The storage systems used by the farmers in preserving their feeds and feedstuffs included in bags, containers, bales and pits, while some practice open grazing systems (field), with 16/21 (76%) of farmers storing their feeds for less than 1 month, 4/21 (19%) keeping their feeds between 3–6 months, and only 1/21 (5%) storing their feeds for more than 6 months (Supplementary Table S1). A total of 65 dairy cattle feeds and feedstuffs (35 in summer and 30 in winter) consisting of silages (n = 4), lucerne (n = 11), pellets (n = 12), grasses (n = 8), total mixed rations (n = 22) and other feeds, including maize stover (n = 1), dairy concentrate (n = 5) and molasses (n = 2) were donated by smallholder dairy cattle farmers from both provinces between summer 2018 and winter 2019 (Supplementary Table S2). The feed samples were collected from the individual participating farmer’s storage system, with one or more representative ingredient batches selected randomly. About 300–500 g/samples were collected from each batch’s lower, middle, and upper layers, mixed thoroughly, placed in sterile plastic bags, kept in cooler boxes, and transported to the University of Johannesburg. Each sample was pulverised using a sterile laboratory blender (LBIOG, ITM Instrument, Edmonton, Alberta, Canada) and stored at −4 °C before fungal enumeration.

### 2.3. Fungal Isolation and Enumeration

The method of fungal isolation and enumeration described by Ekwomadu et al. [23] was adopted in this study with some modifications. Briefly, 1 g of each blended sample was weighed into a sterile test tube containing 9 mL of sterilised Ringer’s salt solution, vortexed, and serially diluted to 10^−6^. Aliquots (1 mL) of each dilution for each sample were inoculated in triplicate on solidified Czapek yeast extract agar (CYA), malt extract agar (MEA), and potato dextrose agar (PDA) (Merck, Darmstadt, Germany), using the spread plate technique. All Petri dishes were supplemented with 100 mg/L streptomycin and chloramphenicol to inhibit bacterial growth. The plates were incubated at 27 °C for 5 to 7 days. Afterwards, the fungal colonies were examined and counted using a colony counter (Gallenkamp, UK). The total and mean fungal loads were calculated and expressed in colony-forming units per gram of sample (CFU/g). Furthermore, the mycological quality was evaluated according to Greco et al. [24]: samples were classified as bad (count range > 7.0 × 10^4^ CFU/g), normal (count range 3.0–7.0 × 10^4^ CFU/g), or good (count range < 3.0 × 10^4^ CFU/g).

(1) $$CFU/g=\frac{Numbers\;of\;colonies\times reciprocal\;of\;the\;dilution\;factor}{plating\;volume\;\left(1\;mL\right)}$$

### 2.4. Conventional Fungal Identification

Each of the different colonies was sub-cultured on a solidified PDA for *Fusarium*, CYA for *Aspergillus*, and MEA for *Penicillium* under aseptic conditions. The plates were wrapped with parafilm and incubated at 27 °C for 5 to 7 days. Thereafter, the fungal morphology was examined macroscopically by observing the appearances (size, colour, shape, texture and aerial hyphae) of the cultures on the media. Microscopic identification was accomplished by placing harvested pure colonies on microscopic slides, stained with lactophenol blue solution, and viewed under the microscope (Olympus CX40, Micro-Instruments News Zealand, Ltd.). Both macro- and microscopic identification of the genera *Fusarium* were done following the taxonomic keys and guides described by Leslie and Summerell [25]. *Penicillium*, *Aspergillus* and other fungal genera were identified according to Klich [26] and Pitt and Hocking [27]. In a situation wherein morphological characteristics of individual fungal isolates following the conventional method were insufficient for precise identification, molecular analysis was performed to determine their identities. The relative density (RD) and isolation frequency (FR) of genus/species were obtained according to Greco et al. [24] as follows:

(2) $$FR\;\left(\%\right)=\frac{number\;of\;samples\;contaminated\;by\;a\;genus\;or\;species\times 100}{total\;number\;of\;samples\;analysed}$$

(3) $$RD\;\left(\%\right)=\frac{number\;of\;isolates\;of\;a\;genus\;or\;species\times 100}{total\;number\;of\;fungi\;isolated}$$

### 2.5. Molecular Identification of Fungal Isolates

Molecular identification of fungi was carried out at the African Centre for DNA Barcoding (ACDB) Lab, University of Johannesburg, South Africa.

#### 2.5.1. Deoxyribonucleic Acid (DNA) Extraction and Polymerase Chain Reaction (PCR) Analysis

Genomic DNA was extracted from each fungal isolate using the Fungal/Bacteria DNA extraction kit (Zymo Research, D6005, Irvine, CA, USA) following the manufacturer’s instructions. Briefly, fungal isolates were subcultured on PDA plates, and pure mycelia from 7-day-old cultures were harvested for DNA extraction. About 150 mg of the harvested mycelia was suspended in 700 µL of lysis solution contained in a 1.5 mL ZR Bashing Bead^TM^ lysis tube. The extracted DNA was quantified using an ND-1000 spectrophotometer (NanoDrop Technologies), adjusted to a working concentration of about 50 ng/µL, and kept at −20 °C prior to further analysis.

DNA fragment of interest within the Internal Transcribed Spacer (ITS) region was amplified by polymerase chain reaction (PCR) after DNA extraction. An amplicon of about 450 bp was obtained from the genomic DNA of the isolates by using the primer combinations ITS-1; 5′- TCC GTA GGT GAA CCT GCG G-3′ (forward) and ITS-4; 5′- TCC TCC GCT TAT GC-3′ (reverse) previously described by White et al. [28]. The PCR was performed using a Red Taq PCR mix (Sigma-Aldrich, Germany). Each sample’s PCR mix consisted of 12.5 µL of PCR mix, 0.5 µL of dimethyl sulfoxide (DMSO), 0.8 µL of DNA sample, 0.3 µL of each primer (ITS1 and ITS4), and 9.6 µL of double-distilled H_2_O to make a final volume of 24 µL. A negative control containing every reagent except for the DNA was also prepared. The PCR was carried out in a ProFlex 32-well PCR system (ThermoFisher Scientific, Singapore) with initial denaturation of DNA set at 95 °C for 2 min, 35 cycles denaturation at 95 °C for 30 secs, an annealing phase at 50 °C for 30 secs, and extension of primer at 72 °C for 1 min. This was followed by a last elongation phase of 10 min at 72 °C, holding for 4 °C. Successful PCR amplifications were confirmed by staining 4 µL of PCR product with 2 µL of ethidium bromide and electrophoresed on 2% agarose gel. The generated bands on the gels were then visualised using a Gel IX imager 20—2.8 M Pixel (Bio Olympics, Thousand Oaks, CA, 33 USA) ultraviolet (UV) transilluminator with a wavelength of 315 nm. Finally, PCR products were purified using a DNA ZR-96 sequencing clean-up kit (Applied Biosystems, Foster City, CA, USA).

#### 2.5.2. DNA Sequencing and Phylogenetic Analysis

The obtained PCR products were sequenced on an ABI 3130 × 1 Genetic Analyzer (ThermoFisher Scientific, Tokyo, Japan) at the African Centre for DNA Barcoding (ACDB) Lab, University of Johannesburg, South Africa. The sequencing reaction mixture contained the same primers as the initial PCR reaction and the BigDye Terminator v. 3.1 Cycle Sequencing Kit (Applied Biosystems, Foster City, CA, USA). Obtained consensus sequences were used to query the GenBank gene sequence database via BLAST: http://www.ncbi.nlm.nih.gov/ (accessed on 14 July 2022) to confirm the presumptive identity of isolates at similarity index scores above 90%. A data set was generated by obtaining the sequences of closely related species to those from this study in the GenBank. The generated DNA sequences were aligned through Muscle, and the aligned sequences were then used to construct phylogenetic trees using MEGA 7.0 [29].

The evolutionary relationship of fungal sequences obtained from the feed samples and their reference strains were generated using the maximum likelihood (ML) method of Tamura and Nei [30]. The bootstrap values based on 1000 replications were used as parameters for the phylogenetic tree construction [31], with all branches corresponding to partitions reproduced lower than 50% of bootstrap replicates collapsed. Finally, the derived phylogenetic trees were used to confirm the evolutionary link between the isolated fungal species from this study and their GenBank relatives. All identified fungal isolates were transferred to PDA slants and preserved in the cultured collection at the University of Johannesburg’s fungal and mycotoxin laboratory, while newly generated sequences were deposited in GenBank.

### 2.6. Statistical Analysis

Data analysis was carried out using IBM Statistical Package for SPSS 22.0 (SPSS^®^ Inc., Chicago, IL, USA). The independent sample *t*-test was used to compare the variation in fungal genera isolation frequencies between the two seasons (summer and winter) at 95% (*p* < 0.05). Shannon–Wiener index (H′) was also used to compare the fungal species biodiversity in the feed sample over the two seasons. The Shannon–Wiener index (H′) values were calculated using MS Excel 2016. This is one of the most common parameters used to quantify and describe the population diversity in different types of samples. In more detail, the Shannon–Wiener index (H′) was calculated as follows:

(H′): −ΣPi × Ln (Pi)(4)

where Σ is a Greek symbol that means “sum”; Pi (ni/N) is relative abundance of the isolated fungal species, N is the total species number of isolates present in each season, ni is the number of isolates of one species and ln is Natural log.

## 3. Results

### 3.1. Fungal Identification

**Figure 1:**
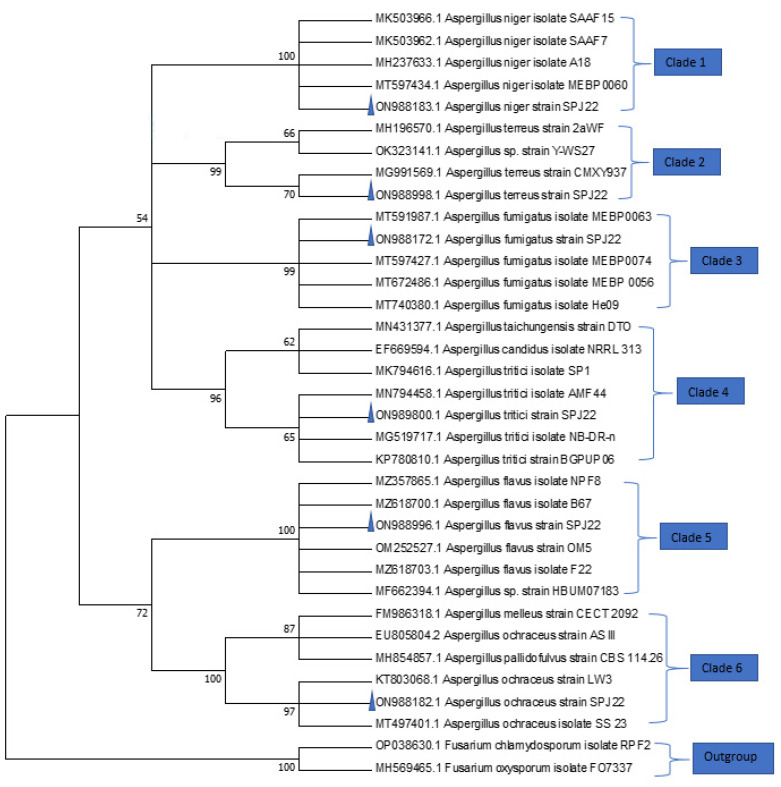
Phylogeny of *Aspergillus* isolates (taxa names with triangles) from dairy feeds based on ITS-region sequence homology. Numbers within the tree represent the bootstrap values of 1000 replicates. The phylogram is rooted (outgroup) with *F. chlamydosporum* and *F. oxysporum*.

**Figure 2:**
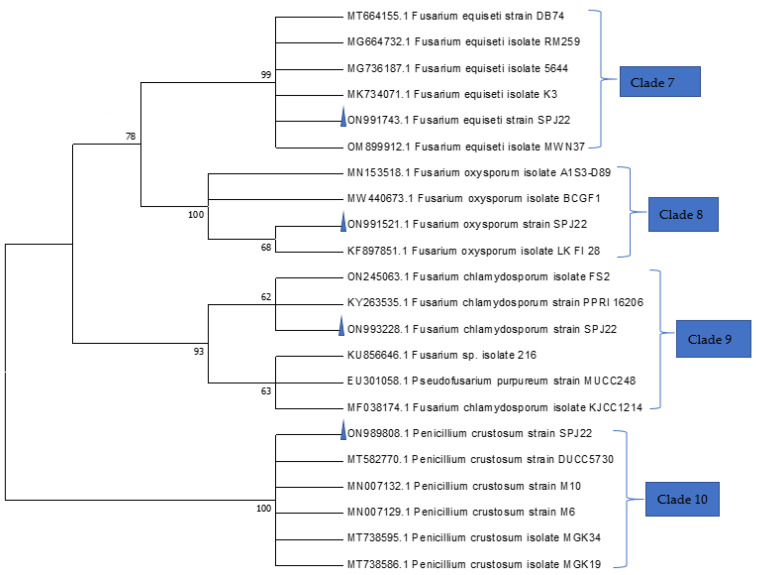
Phylogeny of *Fusarium* and *Penicillium* isolates (taxa names with triangles) from dairy cattle feeds based on ITS-region sequence homology. Numbers within the tree represent the bootstrap values of 1000 replicates.

The fungal isolates were identified using both morphological features and molecular approaches. Their conidia germinated on MEA, CYA and PDA at 27 °C within 7 days. Colour variations were observed in the three media. Based on the phylogenetic analysis, the sequences were grouped into 10 clades (Figure 1 and Figure 2). Isolate ON988183 was grouped in clade 1 with confirmed *A. niger* isolates (MK503966, MK503962, MH237633 and MT597434) with 100% bootstrap value, while ON988998 was grouped with *A. terreus* (MG991569) in clade 2. Isolate ON988172 was associated with *A. fumigatus* isolates in clade 3 with 99% bootstrap value. Furthermore, ON989800 was also found in clade 4 with *A. tritici* isolates (MN794458, MG519717 and KP780810), while ON988999 was associated with *A. flavus* isolates in clade 5 with 100% bootstrap value. The isolate ON988182 was grouped with *A. ochraceus* isolates (KT803068 and MT497401) in clade 6 with 97% bootstrap value. In a similar analysis, isolate ON991743 was grouped in the same clade with *F. equiseti* isolates with 99% bootstrap value. In addition, isolate ON991521 was classified in clade 8 with *F. oxysporum* isolate (KF897851), while isolate ON993228 was grouped with confirmed *F. chlamydosporum* isolates (ON245063 and KY263535) in clade 9. Lastly, isolate ON989818 was associated with confirmed *P. crustosum* isolates in clade 10 with 100% bootstrap value. 

### 3.2. Fungal Contamination

**Figure 3:**
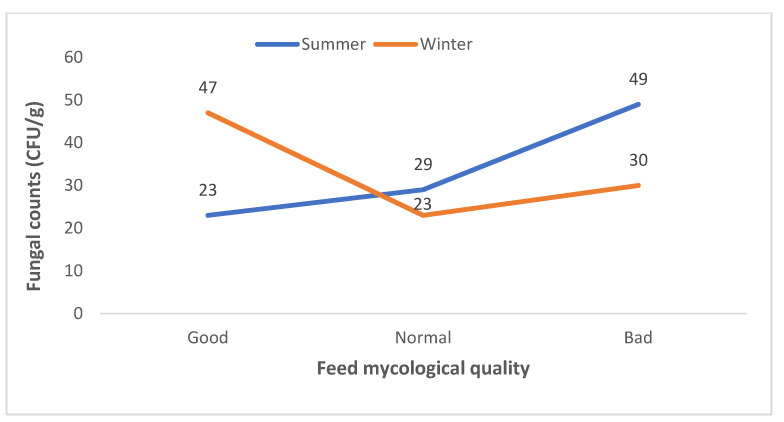
Mycological quality of dairy cattle feeds collected during summer and winter seasons in SA.

In our study, fungal counts (CFU/g) were determined from several dairy cattle feeds and ingredients and the mycological quality criterion revealed that 23% of summer feeds could be classified as good, 29% as regular and 49% as bad, while 47, 23 and 30% of winter feeds could be classified as good, normal and bad, respectively (Figure 3 and Supplementary Table S3).

The range and mean fungal population represented as CFU/g for various feeds collected during the summer and winter seasons are presented in Table 1. Overall, the fungal loads (CFU/g) varied considerably between seasons. A high fungal counts range of 6.1 × 10^3^ to 3.0 × 10^6^ CFU/g was recorded in summer feeds, while winter feeds had fungal load ranging from 1.1 × 10^3^ to 4.1 × 10^5^ CFU/g, respectively. Summer total mixed rations (TMRs) and pellets were the most contaminated feeds, with mean fungal counts of 7.1 and 2.5 × 10^5^ CFU/g. In contrast, the least contaminated samples include winter silages and other feeds sourced during the winter season, with mean fungal counts of 1.9 and 1.2 × 10^4^ CFU/g, respectively.

### 3.3. Fungal Genera and Species Diversity

**Figure 4:**
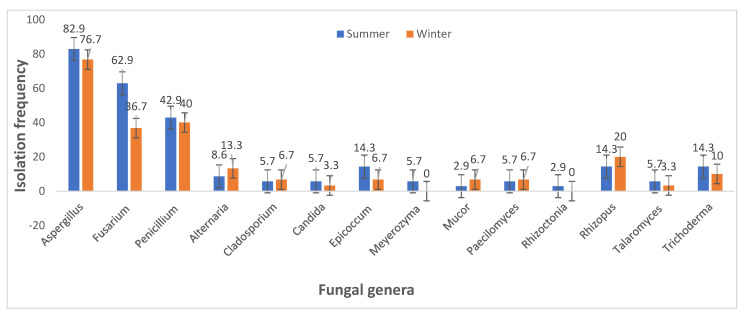
Isolation frequency of fungal genera in dairy cattle feeds and feedstuffs during summer and winter seasons in South Africa.

The data on fungal contamination obtained in the current study (Figure 4 and Supplementary Table S4) revealed the presence of 14 fungal genera. The most predominant genera were *Aspergillus* (80%), whose percentage incidence decreased from summer (82.9%) to winter (76.7%), and *Fusarium* (50.8%), with isolation frequencies of 62.9 and 36.7% in summer and winter, respectively. This was closely followed by *Penicillium* genus (41.5%), with, respectively, 42.9 and 36.7% percentage frequencies in summer and winter samples. As shown in Table 2, the isolation frequencies of the genera *Aspergillus* and *Fusarium* were significantly higher in the summer ((82.867 ± 0.950) and (62.933 ± 1.504)) than winter ((76.667 ± 2.542) and (36.700 ± 2.476)), with *p* ≤ 0.05. In the case of *Penicillium*, its prevalence in summer (42.900 ± 3.418) and winter (40.033 ± 2.967) was not statistically different (*p* ≥ 0.05). Furthermore, the incidence of other genera was significantly different in the two seasons (*p* ≤ 0.05), except for *Cladosporium* and *Paecilomyces* with *p* > 0.05.

The frequency of isolation and relative density of the different fungal species isolated from the feed samples over the two seasons are reported in Table 3. Among the 217 fungal species recovered in this study, 129/217 (59%) of them were recovered from the summer feeds, and 88/217 (41%) from the winter feeds. However, *A. fumigatus* was the most dominant, with a low incidence of 42.8% in the summer feeds and a high incidence of 50.0% in the winter feeds. This was closely followed by *P. crustosum*, which was more prevalent in summer samples (42.9%) than in winter samples (40.0%). *A. flavus* and *A. niger* also were, respectively, recovered at higher incidence rates of 48.6 and 40.0% during the summer compared to 30.0 and 26.7% recorded during the winter. Other less frequently isolated *Aspergillus* spp. found in feeds from both seasons included *A. terreus*, *A. tritici* and *A. ochraceus*.

Furthermore, among *Fusarium* spp., *F. chlamydosporium* and *F. oxysporum* had the highest prevalence, with isolation percentages decreasing from 20.0 and 22.9% in summer to 13.3 and 10.0% in winter, respectively. Moreso, *F. verticillioides*, *F. equiseti* and *F. incarnatum* were also found, albeit at very low frequencies. Interestingly, all five *F. incarnatum* isolates reported in this survey were found in summer feeds.

**Figure 5:**
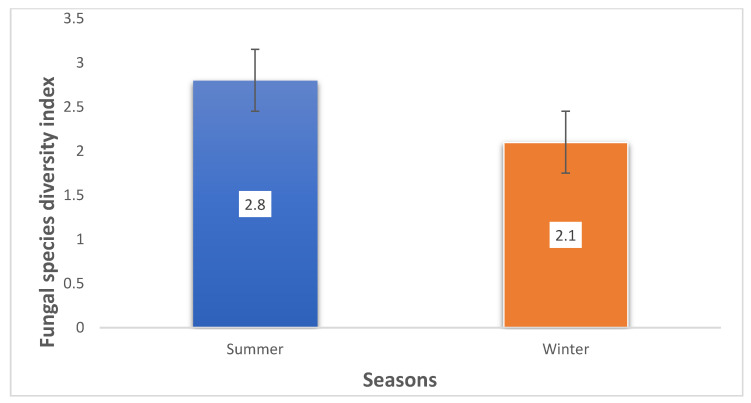
Shannon–Weiner (H′) diversity index of isolated fungal species from SA dairy cattle feed and feed ingredients during summer and winter seasons.

Approximately 97% of the analysed feeds were contaminated with at least one fungus species, with a high co-occurrence of two or more fungal species, notably *A. fumigatus*, *A. flavus*, *A. niger* and *P. crustosum* observed in feeds from both seasons. The Shannon–Weiner diversity index (H′) presented in Figure 5 and Supplementary Table S5 revealed a higher fungal abundance in summer (H′ = 2.8) than in winter (2.1).

## 4. Discussion

Fungal contamination of animal feeds and feed ingredients is a global problem due to their ubiquitous nature and associated toxins (mycotoxins) they produce, which can be detrimental to animal and human health [32] with significant impact on any country’s economy [33]. Fungal contamination is a major contributor to agricultural product losses in SA [11,23] like in most countries in Africa. Therefore, there is a growing demand in the country for a better feed management to assist in monitoring moulds in dairy cattle feeds and feedstuffs throughout the year. This study focused on the incidence and seasonal variability of fungal species associated with feeds destined for smallholder dairy cows in SA in order to provide more information on how to mitigate the threats posed by these mycoflora to dairy cattle, as well as the contamination of milk and milk products by mycotoxins. Many of the analysed feeds, including grasses, lucerne, TMR, pellet, silage, and others such as maize stove, molasses, and dairy concentrates, were found to be contaminated with diverse fungal species with a high degree of seasonal variation.

In the identification study, we used both morphological and molecular techniques. Due to the ubiquity of fungi, as well as their complex and unstable taxonomic history, conventional identification methods must be complemented with molecular techniques for accurate identification [34]. The current phylogenetic analysis based on 16S rRNA genes revealed several new fungal strains in the feed samples. However, mutation and recombination have been identified as some of the causes of genetic diversity in fungal species [35]. The phylogenetic analysis also showed that most of the examined fungal species from the dairy feeds and feedstuffs were closely related to their GenBank relatives.

This study revealed that various storage and field fungi associated with dairy feeds in SA are season dependent. Based on the mycological evaluation, most of the winter feeds were classified as good, while the majority of summer feeds were categorised as bad. Furthermore, higher fungal load was recorded in summer feeds than winter feeds. These findings concur with those of Alam et al. [36], who found that poultry feeds are more susceptible to fungal contamination during summer than in winter. In another study conducted in Brazil, Keller et al. [37] reported the highest mean fungal load in dairy cow feeds (corn and corn meal) cultured on Dichloran Rose Bengal Chloramphenicol (DRBC) agar in summer (5.8 × 10^5^ CFU/g), and the lowest in winter (3.4 × 10^5^ CFU/g) and autumn (4.3 × 10^4^ CFU/g), respectively. Keller et al. [37] also found the highest fungal concentrations in maize silage and wheat brew silage cultured on the same agar in summer (7.3 × 10^5^ CFU/g), whereas winter and spring had the lowest concentrations (7.3 × 10^4^ CFU/g and 1.3 × 10^4^ CFU/g, respectively). According to a study conducted in Norway by Runderget et al. [38], average fungal (*Penicillium*) count in food waste was higher in the summer (6.5 × 10^6^ CFU/g) than in winter samples (1.6 x10^4^ CFU/g). However, this study contradicted that of Ghiasan and Maghsoods in Iran [39], who found that fungal concentrations were higher in winter dairy feeds than in summer feeds.

The low level of fungal contamination detected in silages in this study could be linked to the fermentation process during silage production. According to Adebiyi et al. [40], fermentation aids in inhibiting and suppressing the growth of pathogenic microorganisms, including fungal species. Ndlovu and Dutton [41] also confirmed this by isolating over 100 fungal isolates, including *A. flavus*, *A. fumigatus*, *A. niger*, *A. ochraceus*, *F. sporotrichioides*, *F. graminearum*, *P. expansum*, and *P. citricum*, among others, from 82 corn silage samples and 172 isolates from just 21 chopped maize samples. Njobeh et al. [42] also revealed that fermented food products such as cassava flour and flakes were the least infected by fungal spp. among food products from Cameroon. Notably, pellets and TMR have higher nutrient contents due to their formulation, which comprises minerals, protein feeds, vitamins, grains, cottonseeds and feed additives [43], making them the perfect breeding environment for mycoflora such as *A. flavus*, *A. parasiticus*, *A. fumigatus*, *A. ochraceus*, *A. candidus, F. incarnatum*, *F. solani*, *P. solitum*, *P. citrinum*, *P. chrysogenum* and other fungal spp. [10,44]. This could explain the significant fungal loads found in the TMR and pellets in this investigation. Fungal contamination has been documented in a variety of SA food, including maize [11,23], fermented foods [45], barley and barley products [46], peanut [47], wheat and wheat-based products [48], and animal feeds [41,49,50]. The dominant fungal genera found in this present study during both seasons were *Aspergillus, Fusarium*, and *Penicillium*. Other fungal genera recovered from the feed samples in descending order of preference include *Rhizopus*, *Trichoderma*, *Alternaria*, *Epicoccum*, *Cladosporium*, *Paecilomyces*, *Mucor*, *Candida*, *Talaromyces*, *Meyerozyma* and *Rhizoctonia*. The high prevalences of *Aspergillus*, *Fusarium,* and *Penicillium* identified in this study are similar to those found in previous food and feed investigated in SA [23,41,49]. Saleemi et al. [51] also discovered that *Aspergillus*, *Fusarium* and *Penicillium* were the most abundant fungal genera recovered from maize and maize gluten meal in Pakistan, with isolation frequencies of 33, 11 and 28%, respectively. Similarly, Richard et al. [52] confirmed that these three aforementioned fungal genera are the most occurring contaminants of cattle feeds in France, with a similar report by Ghiasan and Maghsood [39] identifying *Aspergillus* (37.3%), *Penicillium* (23.7%), and *Fusarium* (17.5%) isolated from dairy cattle feed in Iran, although the latter study had higher contamination levels.

Among the *Aspergillus* spp. isolated in this current survey, *A. fumigatus* and *A. flavus*, followed by *A. niger*, were the most prevalent across the two seasons. The prevalence of these three fungal species in both seasons suggests that they can easily adapt to any climatic conditions [53]. The current study’s findings are in line with those of Chilaka et al. [11], who reported *A. fumigatus* (45%), *A. flavus* (43%), and *A. niger* (23%) as the most common *Aspergillus* spp. in SA maize, an important dairy cow feed. A similar study conducted by Ndlovu and Dutton [41] revealed 15 *Aspergillus* spp. in SA dairy cattle feed (maize silage and chopped maize), with *A. fumigatus*, *A. flavus* and *A. niger* being the dominant fungal species occurring at incidence rates of 32, 21 and 11%, respectively. In Iran, Davari et al. [54] reported *A. fumigatus* (21.81%), *A. flavus* (17.27%) and *A. niger* (10%) as the prevalent *Aspergillus* spp. isolated from dairy feedstuffs in the country, while Keller et al. [37] also found that *A. fumigatus*, *A. flavus* and *A. niger* were the among *Aspergillus* spp. contaminating Brazilian corn silage.

Furthermore, *F. chlamydosporum* and *F. oxysporum* were the most frequent species within this genus. This agrees with previous reports that *F. oxysporum* and *F. chlamydosporum* are the most abundant *Fusarium* spp. in SA agricultural products [11,23]. Lastly, *P. crustosum*, the only *Penicillium* spp. recovered in the feed samples, has been documented as the most prevalent *Penicillium* spp. infecting abalone feed in SA [49]. In a study conducted in Cameroon, Njobeh et al. [42] reported *P. crustosum* and *P. polonicum* as the only *Penicillium* spp. recovered from some food samples in the country. Sumarah et al. [55] also revealed the prevalence of *P. crustosum* in samples of grass silage and maize in Canada.

Analysis using the independent sample *t*-test showed significant seasonal differences (*p* > 0.05) in the isolation frequency of the fungal genera detected in this survey, except for *Penicillium*, *Cladosporium* and *Paecilomyces*. The isolation frequencies of fungal genera such as *Aspergillus*, *Fusarium*, *Candida*, *Epicoccum*, *Meyerozyma*, *Rhizoctonia*, *Talaromyces* and *Trichoderma* obtained in the summer period were significantly higher (*p* > 0.05) than in winter. In contrast, *Alternaria*, *Mucor*, and *Rhizopus* were significantly higher in winter (*p* > 0.05) than in summer. This finding corresponds with the findings of Chen et al. [56], who recorded maximum fungal colonisation and abundance rates in Cruciferous crops from Fujian Province, China, in autumn and summer, with the lowest rates observed in winter. These findings are similar to those of González-Jartín et al. [57], who found more *Aspergillus* species in summer dairy cow feeds than in winter in Vila do Conde, north Portugal, while Iram [58] identified the highest proportion of *Aspergillus* species (*A. flavus*) in summer (92.5%) than in winter (40%). Likewise, Chi et al. [59] discovered that *Fusarium* spp. are more prevalent in summer than winter in the Taiwanese mangrove plant *Acanthus ilicifolius var. xiamenensis*.

Finally, the findings reported herein revealed that *Penicillium* (*P. crustosum*) detected did not show any significant seasonal variation; however, more *P. crustosum* was detected in summer samples than in winter samples. According to Hassan [60], *Penicillium* spp. can grow in the autumn, summer and winter seasons. This study is in agreement with the work of Rundberget et al. [38], wherein the same *Penicillium* spp. predominated in both summer and winter samples; nevertheless, the prevalence of *P. crustosum* was higher in the summer (52%) than in the winter samples (12%) in that study. Regarding *Alternaria*, this fungal genus was not the predominant fungi isolated in this study (10.8%), but their higher occurrence in winter (13.3%) compared to summer (8.6%) conforms with the study of Ghiasian and Maghsood [39], who isolated more *Alternaria* spp. (2289) in winter than summer (1153) in Iranian cow feeds.

In terms of fungal species biodiversity, summer feeds have more fungal species (24) than winter feeds (20), with species such as *F. incarnatum*, *Alternaria infectonia*, *Meyerozyma carribica*, and *Rhizoctonia solani* absent in winter feeds. In general, fungal species diversity was shown to be higher in rainy and summer seasons [61], as wetter and warmer conditions promote fungal growth [62]. Our result agrees with De Souza Sebastianes et al. [63], Jahromi et al. [64] and Thompson et al. [65], who recorded higher fungal species diversity in the summer than in the winter in diverse crops. Nonetheless, several studies have documented a greater diversity of fungi in the winter than in the rainy and summer seasons [59,66].

The high concentrations, isolation percentage, and fungal species diversity recorded in summer feeds in this study indicated that fungi thrive more in summer than winter, as the weather conditions (relative humidity, water activity and temperature) during this period favour the growth of microbes, including fungi [36]. It is important to note that these conditions are not the same for all fungi; they vary depending on the fungus [27]. It has been confirmed that most *Aspergillus* spp. require a higher temperature range (15 to 40 °C) for growth than *Penicillium* spp. (25 to 30 °C); however, the optimum temperature range for *Aspergillus* development is between 24 to 30 °C and 27 °C for most *Penicillium* [27,67]. Unlike the genera *Penicillium* and *Aspergillus*, *Fusarium* spp. may grow at lower temperatures (20 to 30 °C), with 25 °C being the optimum temperature [68]. It has also been established that the maximum growth of storage fungi, especially *Aspergillus* and *Penicillium* spp., occurs between 83 and 85% relative humidity, respectively [27]. According to Cao et al. [69], relative humidity between 25 to 85% significantly promotes the growth of *Fusarium.*

The heavy contamination of the summer feeds could also be linked to post-harvest conditions, including improper feed handling, poor storage facilities and conditions, and transportation methods [70]. Small-scale dairy farmers lack adequate storage facilities to keep their feeds. This was the case during sampling when some of the dairy farmers stored their cattle’s feeds in unhygienic environments and under conditions that promote fungal proliferation. Due to feed shortage in the summer, a large number of feeds (cereals and forages) produced in the winter period are stored until summer in conditions conducive to fungal growth, such as the interaction between O_2_, CO_2_ concentration, and moisture content [27,71]. *Aspergillus* and *Penicillium* are storage fungi [26]; that is, they infest agricultural commodities during storage if storage conditions are not adequately controlled [70]. This may explain the increase in *Aspergillus* and *Penicillium* spp. identified in the summer samples of this investigation.

The late harvesting method employed by the farmers during the summer season may possibly account for the higher fungal contamination levels, isolation frequency and species diversity observed in summer feeds than winter feeds. The summer feeds and feedstuffs, particularly cereals, were left too long on the field before harvesting. Late harvesting of agricultural products has been linked to high contamination of feed and food with fungal species, particularly *Fusarium* spp. in the field [70]. However, some *Fusarium* spp. are able to persist in harvested and store agricultural products and proliferate during storage when moisture content is favourable [72]. Additionally, it is important to note that insect and pest infestation of the feed ingredients on the field could also explain the greater fungal levels, isolation percentage, and fungal diversity recorded during the summer as compared to the winter [73]. As reviewed by Munkvold [74], long duration of feed ingredients in the field allows pests and insects to infect and cause wounds on them via their feeding patterns; these wounds, according to Fandohan [72], could expose the feeds to fungal contamination.

Most of the fungal species recovered in this survey are widely recognised as mycotoxin producers in food and feed [11,41]. *A. flavus* produces aflatoxins, the most notorious group of mycotoxins that are teratogenic, carcinogenic, and immunosuppressive, and which have been linked to chronic carcinogenicity, acute toxicity, and death in animals and humans [32]. *A. Fumigatus*, the most abundant fungal species in this study, is known for its potential to produce trypacidin, a mycotoxin confirmed to be cytotoxic to human lung cells [75]. Furthermore, *F. verticillioides*, the principal fumonisin B_1_ (FB_1_) producer, was recovered from some of the feeds in both seasons. A high incidence of human oesophageal cancer in the Transkei region (now Eastern Cape) in SA was linked to the contamination of maize by FB_1_ in the area [76]. Furthermore, zearalenone (ZEN) which is commonly formed by *F. oxysporum*, *F. incarnatum* and *F. equiseti*, causes infertility issues in animals, including dairy cattle [77]. Hence, the co-occurrence of toxigenic fungi, as reported in this study, shows how dairy cattle are exposed to fungal toxins (mycotoxins), with subsequent transfer to humans via consumption of milk, meat and by-products from animals that have fed on such contaminated feeds.

## 5. Conclusions

In conclusion, the current study investigated the hypothesis that differences exist between the dairy cattle feeds and feedstuffs used during the summer and winter seasons in SA regarding fungal contamination and diversity. This investigation further discovered that different fungi (storage and field) are associated with dairy feeds in the country. However, the presence of toxigenic fungi in the feeds does not necessarily imply that mycotoxins naturally occur in them; instead, it notifies us of the potential risk of contamination. Furthermore, the high isolation frequency and fungal diversity, particularly in summer, revealed by this study emphasises the importance of continued research on dairy feed safety with regard to toxigenic fungi contamination in different parts of SA and during different seasons. It is important to mention that certain conditions such as improper feed storage, poor agricultural practices, infestation by pests and insects and climatic conditions may be responsible for the higher fungal contamination levels recorded in summer than winter. As a result, possible measures, including proper storage facilities, good storage conditions, and good agricultural practices, must be implemented to limit health effects on dairy cattle and improve the quality of animal by-products.

## Figures and Tables

**Table 1:** Mean fungal loads and fungal genera recovered from smallholder dairy cattle feeds and feedstuffs from Free State and Limpopo provinces, South Africa.

Feed Samples	Season	+ve Sample	CFU/g Range	Mean	Fungal Genera
Grasses	Summer	7	1.7 × 10^4^–1.6 × 10^5^	9.2 × 10^4^	*Aspergillus, Fusarium, Epicoccum, Penicillium* and *Mucor.*
	Winter	1	1.1 × 10^4^–9.2 × 10^4^	5.2 × 10^4^	*Aspergillus* and *Epicoccum.*
Lucerne	Summer	6	1 × 10^4^–1.3 × 10^5^	6.8 × 10^4^	*Aspergillus, Alternaria, Penicillium, Fusarium, Rhizopus, Talaromyces* and *Trichoderma.*
	Winter	5	2 × 10^4^–4.1 × 10^5^	1.4 × 10^5^	*Aspergillus, Epicoccum, Fusarium* and *Trichoderma.*
Pellet	Summer	4	3 × 10^4^–6.0 × 10^5^	2.5 × 10^5^	*Aspergillus, Penicillium, Rhizopus, Rhizoctonia, Talaromyces* and *Fusarium.*
	Winter	7	1.1 × 10^3^–9.0 × 10^4^	2.9 × 10^4^	*Aspergillus, Alternaria, Fusarium, Penicillium, Mucor, Rhizopus, Talaromyces* and *Trichoderma.*
Silage	Summer	1	9.2 × 10^3^–3.5 × 10^4^	2.1 × 10^4^	*Fusarium* and *Yeast.*
	Winter	2	7 × 10^3^–3.0 × 10^4^	1.9 × 10^4^	*Aspergillus, Penicillium, Paecilomyces* and *Yeast.*
TMR	Summer	11	1.2 × 10^4^–3.0 × 10^6^	7.1 × 10^5^	*Aspergillus, Alternaria, Fusarium, Epicoccum, Cladosporium, Penicillium, Paecilomyces, Trichoderma, Rhizopus* and *Yeast.*
	Winter	11	1 × 10^4^–2.4 × 10^5^	1.2 × 10^5^	*Aspergillus, Alternaria, Fusarium, Penicillium, Cladosporium, Mucor, Trichoderma* and *Rhizopus.*
Others	Summer	6	6.1 × 10^3^–5.0 × 10^5^	2.1 × 10^4^	*Aspergillus, Fusarium, Cladosporium, Penicillium, Trichoderma* and *Yeast.*
	Winter	2	1.1 × 10^4^–1.3 × 10^4^	1.2 × 10^4^	*Aspergillus, Alternaria, Fusarium, Penicillium, Paecilomyces* and *Rhizopus.*

Others = dairy concentrates (5), maize stover (1), molasses, and (2) TMR = Total Mixed Ration; CFU/g = Colony forming unit per gram of sample; No = Number.

**Table 2:** Independent sample *t*-test on variation with isolation frequencies of fungal genera recovered from dairy cattle feeds during summer and winter seasons in Free State and Limpopo provinces, SA.

Fungal Genera	Seasons	Mean ± SD	T	DF	Sig.
*Aspergillus*	Summer	82.867 ± 0.950	3.957	4	0.017
	Winter	76.667 ± 2.542			
*Fusarium*	Summer	62.933 ± 1.504	15.684	4	0.000
	Winter	36.700 ± 2.476			
*Penicillium*	Summer	42.900 ± 3.418	1.097	4	0.334
	Winter	40.033 ± 2.967			
*Alternaria*	Summer	8.600 ± 0.854	−5.192	4	0.007
	Winter	13.267 ± 1.301			
*Cladosporium*	Summer	5.667 ± 1.060	−1.592	4	0.187
	Winter	6.733 ± 0.473			
*Candida*	Summer	5.667 ± 1.069	2.811	4	0.048
	Winter	3.333 ± 0.961			
*Epicoccum*	Summer	14.333 ± 0.851	8.331	4	0.001
	Winter	6.733 ± 1.332			
*Mucor*	Summer	2.867 ± 0.833	−5.549	4	0.005
	Winter	6.733 ± 0.874			
*Paecilomyces*	Summer	5.733 ± 0.252	−1.577	4	0.190
	Winter	6.733 ± 1.069			
*Rhizopus*	Summer	14.333 ± 1.1676	−3.858	4	0.018
	Winter	20.000 ± 2.2605			
*Talaromyces*	Summer	5.733 ± 0.815	3.437	4	0.026
	Winter	3.300 ± 0.917			
*Trichoderma*	Summer	14.333 ± 1.1021	3.437	4	0.026
	Winter	10.033 ± 1.021			

SD = standard deviation; DF = degree of freedom; T = t-value.

**Table 3:** Isolation frequency and relative density of fungal species recovered from smallholder dairy cattle feeds and feed ingredients in Free State and Limpopo provinces, SA.

Season	Summer (35)			Winter (30)			
*Aspergillus* spp.	No. of Iso.	FR (%)	RD (%)	No. of Iso.	FR (%)	Rd (%)	Accession No.
*A. flavus*	17	48.6	13.2	9	30	10.2	ON988996
*A. fumigatus*	15	42.8	11.6	15	50	17.0	ON988172
*A. niger*	14	40	10.9	8	26.7	9.1	ON988183
*A. ochraceus*	1	2.9	0.8	1	3.3	1.1	ON988182
*A. terreus*	6	17.1	4.7	5	16.7	5.7	ON988998
*A. tritici*	2	5.7	1.6	2	6.7	2.3	ON989800
***Penicillium* spp.**							
*P. crustosum*	15	42.9	11.6	12	40	13.6	ON989808
***Fusarium* spp.**							
*F. chlamydosporum*	7	20	5.4	4	13.3	4.5	ON993228
*F. equiseti*	3	8.6	2.3	4	13.3	4.5	ON991743
*F. oxysporum*	8	22.9	6.2	3	10	3.4	ON991521
*F. incarnatum*	5	14.3	3.9	-	-	-	
*F. verticillioides*	6	17.1	4.7	2	6.7	2.3	
**Other spp.**							
*Epicoccum sorghinum*	5	14.3	3.9	2	6.7	2.3	ON994254
*Paecilomyces maximus*	2	5.7	1.6	2	6.7	2.3	ON989798
*Talaromyces pinophilus*	2	5.7	1.6	1	3.3	1.1	ON598611
*Alternaria alternata*	1	2.9	0.8	4	13.3	4.5	
*Alternaria infectoria*	2	5.7	1.6	-	-	-	
*Candida albican*	2	5.9	1.6	1	3.3	1.1	
*Cladosporium cladosporioides*	2	5.9	1.6	2	6.7	2.3	
*Meyerozyma carribica*	2	5.9	1.6	-	-	-	
*Mucor plumbeus*	1	2.9	0.8	2	6.7	2.3	
*Rhizoctonia solani*	1	2.9	0.8	-	-	-	
*Rhizopus solonifer*	5	14.3	3.9	6	20	6.8	
*Trichoderma atroviride*	5	14.3	3.9	3	10	3.4	
***Total***	129			88			

FR = isolation frequency; RD = isolation relative density; No. = number, and Iso. = Isolation.

## Data Availability

Data will be provided upon request.

## Supplementary Materials

The following supporting information can be downloaded at: https://www.mdpi.com/article/10.3390/jof8111192/s1, Table S1: Dairy farms visited, provinces, number of feeds collected in each season, storage method employed by the farmers and the duration of storage; Table S2: Dairy cattle feeds and feed ingredients donated by different smallholder dairy farmers in Free State and Limpopo provinces of SA and during the winter and summer seasons between 2018 to 2019; Table S3: Dairy cattle feeds and feed ingredients obtained from Free State and Limpopo provinces in SA, the fungal load, season and mycological quality; Table S4: Isolation frequency of fungal genera recovered from dairy cattle feeds and feedstuffs from Free State and Limpopo provinces of SA during summer and winter seasons; Table S5: The Shanon-Weiner diversity index (H’) showing fungal species abundance in summer and winter dairy cattle feeds.
