# Supplementary material for: Seasonal Diversity and Occurrence of Filamentous Fungi in Smallholder Dairy Cattle Feeds and Feedstuffs in South Africa
Source: J Fungi (Basel). 2022 Nov 11;8(11):1192. doi: 10.3390/jof8111192 (PMC9696519; doi:10.3390/jof8111192)
Supplement: Supplementary file 1 [file jof-08-01192-s001.zip › jof-1998657-supplementary.pdf]

Supplementary Table S1. Dairy farms visited, provinces, number of feeds collected in each season, storage method employed by the farmers and the duration of storage.

Farms	Province	No of feedscollected in summer	No of feeds collected in winter	Storage method	Storage duration
Farm 1	Free State	2	1	Bags	< 1 month
Farm 2	Free State	1	2	Container, field	3 – 6 months
Farm 3	Free State	3	1	Bales, field	< 1 month
Farm 4	Free State	2	2	Bale, field	< 1 month
Farm 5	Free State	3	1	Bags	< 1 month
Farm 6	Free State	2	2	Container	3 – 6 months
Farm 7	Free State	1	2	Bale, field	< 1 month
Farm 8	Free State	1	1	Pit, bags	< 1 month
Farm 9	Free State	2	2	Pit, field	< 1 month
Farm 10	Free State	1	2	Container	< 1 month
Farm 11	Free State	1	2	Container	> 6 months
Farm 12	Limpopo	2	1	Bales, field	< 1 month
Farm 13	Limpopo	2	1	Pit, field	< 1 month
Farm 14	Limpopo	2	2	Bags, bales	< 1 month
Farm 15	Limpopo	2	2	Container	< 1 month
Farm 16	Limpopo	1	1	Bags, container	3 – 6 months
Farm 17	Limpopo	1	1	Bales, field	< 1 month
Farm 18	Limpopo	2	1	Container	< 1 month
Farm 19	Limpopo	1	-	Pit, field	3 – 6 months
Farm 20	Limpopo	1	2	Bags, bales	< 1 month
Farm 21	Limpopo	2	1	Bags	< 1 month

< = less than; > = greater than.

Supplementary Table S2. Dairy cattle feeds and feed ingredients donated by different smallholder dairy farmers in Free State and Limpopo provinces of SA and during the winter and summer seasons between 2018 to 2019.

Feed type	Summer	Winter	Total
Grasses	7	1	8
Lucerne	6	5	11
Pellet	4	8	12
Silage	1	3	4
TMR	11	11	22
Others[a]	6	2	8
Total	35	30	65

a = dairy concentrates (5), maize stover (1) and molasses (2).

Supplementary Table S3. Dairy cattle feeds and feed ingredients obtained from Free State and Limpopo provinces in SA, the fungal load, season and mycological quality.

S/N	Sample I.D	Fungal source	Fungal load	Season	Feed mycological quality
1	GF03	Grass	1.7×10^4	Summer	Good
2	GF04	Grass	4.0×10^4	Summer	Normal
3	GF05	Lucerne	1.0×10^4	Summer	Good
4	GF06	Lucerne	1.0×10^5	Summer	Bad
5	GF08	Lucerne	1.2×10^5	Winter	Bad
6	GF09	Grass	5.2×10^4	Winter	Normal
7	GF10	Lucerne	8.0×10^4	Winter	Bad
8	HS01	TMR	1.2×10^4	Summer	Normal
9	HS02	TMR	5.0×10^4	Summer	Normal
10	HS03	TMR	1.7×10^5	Summer	Bad
11	HS04	TMR	2.3×10^5	Summer	Bad
12	HS05	Dairy concentrate	1.0×10^4	Summer	Good
13	HS06	Grass	1.6×10^5	Summer	Bad
14	HS07	TMR	2.8×10^6	Summer	Bad
15	HS08	TMR	2.0×10^4	Summer	Good
16	HS09	TMR	3.0×10^6	Summer	Bad
17	HS10	TMR	3.7×10^5	Summer	Bad
18	HS11	TMR	8.0×10^4	Winter	Bad
19	HS12	Pellet	6.0×10^4	Winter	Normal
20	HS13	TMR	4.0×10^4	Winter	Normal
21	HS14	TMR	1.9×10^4	Winter	Good
22	HS15	TMR	2.0×10^4	Winter	Good
23	HS16	Lucerne	4.1×10^5	Winter	Bad
24	HS17	TMR	1.1×10^5	Winter	Bad
25	HS18	TMR	1.4×10^5	Winter	Bad
26	HS19	Dairy concentrate	1.1×10^4	Winter	Good
27	HS20	Lucerne	5.0×10^4	Winter	Normal
28	HS21	TMR	5.2×10^4	Winter	Normal
29	HS22	TMR	1.0×10^4	Winter	Good
30	HS23	Silage	7.0×10^3	Winter	Good
31	HS24	Silage	nd	Winter	Good
32	HS25	Silage	3.0×10^4	Winter	Normal
33	HS26	TMR	2.4×10^5	Winter	Bad
34	JF01	Pellet	1.9×10^5	Summer	Bad
35	JF02	Grass	2.3×10^4	Summer	Good
36	JF04	Lucerne	2.0×10^4	Summer	Good
37	JF05	Pellet	6.0×10^5	Summer	Bad
38	JF06	Pellet	9.0×10^3	Winter	Good
39	JF07	Pellet	1.7×10^3	Winter	Good
40	JF08	Pellet	1.1×10^3	Winter	Good
41	JF09	Pellet	3.4×10^4	Winter	Normal
42	NJ01	Grass	5.0×10^4	Summer	Normal
43	NJ02	Pellet	3.0×10^4	Summer	Normal
44	NJ03	Lucerne	3.0×10^4	Summer	Normal

45	NJ04	Diary concentrate	1.7×10^5	Summer	Bad
46	NJ05	Silage	2.1×10^4	Summer	Good
47	NJ06	Grass	4.0×10^4	Summer	Normal
48	NJ07	Lucerne	1.3×10^5	Summer	Bad
49	NJ08	Pellet	1.8×10^5	Summer	Bad
50	NJ09	Pellet	1.0×10^4	Winter	Good
51	NJ10	Pellet	nd	Winter	Good
52	NJ11	Pellet	9.0×10^4	Winter	Bad
53	PD02	Grass	1.2×10^5	Summer	Bad
54	PD03	Molasses	4.0×10^4	Summer	Normal
55	PD04	Lucerne	1.2×10^5	Summer	Bad
56	PD05	Maize stove	6.1×10^3	Summer	Good
57	PD06	Dairy concentrate	4.0×10^4	Summer	Normal
58	PD07	Dairy concentrate	5.0×10^5	Summer	Bad
59	PD08	TMR	5.0×10^4	Summer	Normal
60	PD09	TMR	8.0×10^5	Summer	Bad
61	PD10	TMR	3.0×10^6	Summer	Bad
62	PD11	Lucerne	2.0×10^4	Winter	Good
63	PD12	TMR	9.0×10^4	Winter	Bad
64	PD13	Molasses	1.3×10^4	Winter	Good
65	PD14	TMR	2.1×10^4	Winter	Good

nd = not detected.

Supplementary Table S4. Isolation frequency of fungal genera recovered from dairy cattle feeds and feedstuffs from Free State and Limpopo provinces of SA during summer and winter seasons.

S/N	Fungal genus	Summer	Winter	Total
	Aspergillus	29 (82.9)	23 (76.7)	52 (80)
2	*Fusarium*	22 (62.9)	11 (36.7)	33 (50.8)
3	*Penicillium*	15 (42.9)	12 (40)	27 (41.5)
4	*Alternaria*	3 (8.6)	4 (13.3)	7 (10.8)
5	*Cladosporium*	2 (5.7)	2 (6.7)	4 (6.2)
6	*Candida*	2 (5.7)	1 (3.3)	3 (4.6)
7	*Epicoccum*	5 (14.3)	2 (6.7)	7 (10.8)
8	*Meyerozyma*	2 (5.7)	-	2 (3.1)
9	*Mucor*	1 (2.9)	2 (6.7)	3 (4.6)
10	*Paecilomyces*	2 (5.7)	2 (6.7)	4 (6.2)
11	*Rhizoctonia*	1 (2.9)	-	1 (1.6)
12	*Rhizopus*	5 (14.3)	6 (20)	11 (16.9)
13	*Talaromyces*	2 (5.7)	1 (3.3)	3 (4.6)
14	*Trichoderma*	5 (14.3)	3 (10)	8 (12.3)

Supplementary Table S5. The Shanon-Weiner diversity index (H′) showing fungal species abundance in summer and winter dairy cattle feeds.

S/N	*Fungal species*	Summer	H values	Winter	H values
1	*A. tritici*	2	−0.0646	2	−0.086
2	*A. flavus*	17	−0.26707	9	−0.23319
3	*A. fumigatus*	15	−0.2502	15	−0.30158
4	*A. niger*	14	−0.24101	8	−0.21799
5	*A. ochraceus*	1	−0.03767	1	−0.05088
6	*A. terreus*	6	−0.1427	5	−0.16295
7	*P. crustosum*	15	−0.2502	12	−0.2717
8	*F. chlamydosporium*	7	−0.15812	4	−0.1405
9	*F. equiseti*	3	−0.08747	4	−0.1405
10	*F. oxysporum*	8	−0.17243	3	−0.11518
11	*F. incarnatum*	5	−0.12598	0	0
12	*F. verticillioides*	6	−0.1427	2	−0.086
13	*E. sorghinum*	5	−0.12598	2	−0.086
14	*P. maximus*	2	−0.0646	2	−0.086
15	*T. pinophilus*	2	−0.0646	1	−0.05088
16	*A. alternata*	1	−0.03767	4	−0.1405
17	*A. infectonia*	2	−0.0646	0	0
18	*Candida albican*	2	−0.0646	1	−0.05088
19	*C. cladosporioides*	2	−0.0646	2	−0.086
20	*M. carribica*	2	−0.0646	0	0
21	*Mucor*	1	−0.03767	2	−0.086
22	*R. solani*	1	−0.03767	0	0
23	*R. solonifer*	5	−0.12598	6	−0.18311
24	*T. atroviride*	5	−0.12598	3	−0.11518
	Total	129	2.81873	88	2.07027
